# Obituary

**Published:** 1845-10-01

**Authors:** 


*Obituary.*—Dr. Wm. H. Richardson, late professor of obstetrics in the medical department of Transylvania University, died at Caneland, near Lexington, on Sunday evening last. Prof. Richardson stood at the head of his profession in this State, in his particular department, and has been long and favorably known as one of the soundest and best teachers of his section of medical science in this country.—*Louisville Jour.* 18*th*.

